# Longitudinal Association Between Mindfulness and Wisdom: A Follow-Up Study in Emerging Adulthood

**DOI:** 10.3390/jintelligence13090122

**Published:** 2025-09-19

**Authors:** Yimeng Wang, Hao Cheng

**Affiliations:** 1 School of Psychology, Northwest Normal University, Lanzhou 730070, China; wym5852@nwnu.edu.cn; 2 School of Psychology, Nanjing Normal University, Nanjing 210023, China

**Keywords:** mindfulness, wise reasoning, wise thinking, longitudinal design, random intercept cross-lagged

## Abstract

While theoretical frameworks posit mindfulness as a catalyst for wisdom development, longitudinal evidence remains scarce. This study examines the developmental trajectory of wisdom during emerging adulthood and investigates the intra-person and within-person effects of mindfulness on wisdom through a three-wave longitudinal design. A sample of 719 Chinese first-year college students completed assessments of the Five Facet Mindfulness Questionnaire, the Situated Wise Reasoning Scale, and the Wise Thinking Scale across three timepoints. Longitudinal multilevel analysis (LMA) and random intercepts cross-lagged panel models (RI-CLPMs) were employed to distinguish between stable individual differences and temporary fluctuations. Three key findings emerged: (1) Both wise reasoning and wise thinking exhibited linear growth trajectories. (2) At the between-person level, dispositional mindfulness showed strong positive associations with wisdom. (3) Within-person analyses revealed that mindfulness fluctuations prospectively predicted changes in wise reasoning and thinking, establishing temporal precedence. This study provides new evidence that wisdom can be both a developing ability and a stable trait during emerging adulthood. The observed dynamic links between mindfulness and wisdom highlight the potential of mindfulness-based interventions to foster the growth of wisdom.

## 1. Introduction

Wisdom gradually emerges and develops as individuals accumulate life experiences, moral reasoning, and attitudes toward life (Baltes and Staudinger 2000). It can be viewed both as a state and as a comprehensive psychological quality and may also refer to a specific behavior (Wang and Zheng 2022, pp. 79–104). Most empirical investigations of wisdom rely on cross-sectional designs, predominantly assessing state-level manifestations of wisdom rather than examining its long-term developmental trajectories (Fu et al. 2021; Grossmann 2017). Emerging adulthood is a crucial stage in budding wisdom (Jordan 2005). Emerging adulthood is characterized by significant psychological flux, during which wisdom development may exhibit trait-like stability and dynamic context-sensitive variability. This suggests that targeted interventions during this developmental period hold promise in facilitating wisdom cultivation. Recent theoretical frameworks have conceptualized wisdom as a developable construct (Karunamuni and Weerasekera 2019; Verhaeghen 2019), with empirical evidence suggesting mindfulness as a potential facilitator of wisdom development (Sharma et al. 2017; Wang et al. 2022, 2023, 2024). Consequently, elevated or progressively increasing mindfulness levels during early adulthood may significantly accelerate the development of wisdom. This study focused on emerging adulthood as a critical developmental window for accelerated wisdom maturation. Employing a longitudinal design, we systematically examined wisdom’s developmental trajectory while accounting for its dynamic growth processes and stable trait-like characteristics by precisely delineating the initial stages of wisdom formation and mapping its progression during early adulthood. This provided crucial insights into the facilitative role of mindfulness in wisdom development. These findings offer significant theoretical and practical implications for optimizing wisdom education paradigms.

### 1.1. Wisdom

Modern psychology has been exploring theories and empirical research on wisdom for nearly 50 years. Sternberg and Glück (2022), in Wisdom: The Psychology of Wise Thoughts, Words, and Deeds, provide a comprehensive theoretical framework and methodology for the study of wisdom. They divide nearly 50 years of modern wisdom psychology into three stages, noting that in the current stage, wisdom is increasingly regarded as more important than knowledge. This period has seen the emergence of diverse concepts and theories of wisdom and the rise in multiple research hotspots, representing a “flourishing” phase in the field (Zhang et al. 2023). Although wisdom is conceptualized in diverse ways, research indicates that it extends beyond mere knowledge or cognitive processes, encompassing behaviors that benefits oneself, others, and society (Wang and Zheng 2022, p. 203). In particular, Sternberg’s Balance Theory posits that wisdom is not merely the simple application of knowledge or intelligence. Rather, it requires balancing multiple dimensions, including personal interests, the interests of others, higher-order interests (such as those of society or humanity), short-term versus long-term considerations, and the pursuit of common good through positive ethical values (Sternberg and Glück 2022). This theoretical framework provides a foundation for a multidimensional understanding and study of wisdom.

Contemporary empirical research has primarily focused on the cognitive and knowledge-oriented aspects of wisdom (e.g., Brienza et al. 2018; Fu et al. 2021; Grossmann and Kross 2014; Wang et al. 2023). Western researchers have proposed the concept of wise reasoning and argued that wisdom involves practical reasoning that helps individuals address challenges inherent in social life, such as conflicts between groups and individuals (Grossmann 2017). This includes intellectual humility, recognition of others’ perspectives or broader contexts than the issue at hand, recognition of uncertainty and change, and compromise. Based on China’s cultural background, Chinese researchers have proposed the integration theory of virtue and wisdom, defining wisdom through problem-solving ability. They view wisdom as the psychological characteristic of an individual exemplified in solving dilemmas or ambiguous problems, encapsulated in the concept of “moral virtue and smartness” or Wise Thinking (Fu et al. 2021). This concept captures the characteristics of wisdom in problem-solving capabilities expressed through the cognitive processes involved. It highlights the dynamic expression of wisdom in addressing problems and recognizing uncertainty, considering different perspectives, benevolence, and insights (Fu et al. 2021).

Both wise reasoning and wise thinking examine the manifestation of wisdom in complex situations, but they differ in cultural background and theoretical foundations. Wise reasoning is rooted in the Western cognitive tradition of psychology (Wang and Zheng 2022, pp. 111–12), emphasizing practical reasoning skills and grounded in scientific evidence. Theoretically, it draws primarily on cognitive science and social psychology, focusing on how individuals solve problems through logical reasoning and perspective-taking (Grossmann 2017). In contrast, wise thinking is grounded in Chinese traditional culture, particularly the Confucian and Daoist notion of ren’ai (benevolence), emphasizing the integration of virtue and talent (Fu 2019), and highlighting that wisdom encompasses not only cognitive abilities but also moral character. Furthermore, existing studies have introduced wise reasoning measurement scales developed in Western cultural contexts to Chinese samples, revealing that the Chinese version of the scale demonstrates good measurement characteristics among university student samples (Wang et al. 2022, 2023, 2024; Wei and Wang 2021). Therefore, considering that both emphasize the dynamic characteristics of wisdom and align with the current research focus on the dynamic development of wisdom, this study aimed to simultaneously examine the performance of wise reasoning and wise thinking in ambiguous interpersonal conflict issues (Brienza et al. 2018; Fu et al. 2021) to avoid potential confounding effects from measurement tools.

### 1.2. Mindfulness and Wisdom

Mindfulness originates from Buddhist meditation, emphasizing a quiet, introspective method of regulating the body and mind. Kabat-Zinn (2003) stripped it of its religious connotations, combined it with Vipassana meditation, and operationally defined mindfulness as a purposeful, non-judgmental awareness of the present moment. Currently, three main concepts represent mindfulness (Xu 2024, pp. 16–17): (1) trait mindfulness, which views mindfulness as a relatively stable psychological construct similar to personality; (2) state mindfulness, which considers mindfulness as a state of being; and (3) mindfulness interventions or mindfulness training, referring to practices based on mindfulness, such as breath awareness, body scanning, and mindful walking. This study primarily focused on the trait aspect of mindfulness, exploring how mindfulness, as a stable psychological trait, influences individuals’ wise reasoning and wise thinking over time.

Mindfulness and wisdom are multifaceted constructs encompassing multiple dimensions. Scholars have proposed diverse theories and models to explain how mindfulness promotes or predicts the development of wisdom. Synthesizing these perspectives, three key mechanisms appear to underlie their association. The first concerns changes in self-reflection and self-awareness. Mindfulness fosters reflective awareness and self-transcendence, enabling individuals to move beyond subjectivity and cultivate wisdom through ongoing self-examination and perspective-taking. Reflective awareness and self-transcendence are central to the cultivation of wisdom within mindfulness (Verhaeghen 2019). Ardelt (2004) explicitly states that developing wisdom requires transcending subjectivity and projections. Further, mindfulness can aid in cultivating traits of humility and self-transcendence by promoting the ability to adopt a bystander’s perspective or perspective-taking (Desbordes et al. 2015). This ability can guide individuals in gaining deeper insights into the essence of things (Ardelt 2008), thereby facilitating the development of wisdom (Wang et al. 2023, 2024).

The second mechanism is ethical sensitivity (Verhaeghen 2019; Verhaeghen and Aikman 2019). Haidt’s Moral Foundations Theory (MFT; Graham et al. 2011, 2013) posits that there are five moral foundations universally present in humanity: harm or care, fairness or reciprocity, in-group or loyalty, authority or respect, and purity or sanctity. Reflective awareness of mindfulness is directly related to personal moral dimensions (i.e., promoting care and fairness) and self-transcendence is associated with social and moral dimensions (i.e., emphasizing loyalty, respect or authority, and purity or sanctity). Most theories and empirical research in the psychology of wisdom suggest a close relationship between wisdom and good character, particularly manifested in prosocial attitudes and behaviors. These prosocial traits include empathy, compassion, warmth, altruism, and a sense of fairness (Bangen et al. 2013; Verhaeghen and Aikman 2019).

The third mechanism is psychological distance (Kross and Grossmann 2012), which can be explained using the Construal Level Theory (CLT). This theory posits that psychological distance influences psychological processing by affecting the representation of things (Trope and Liberman 2010). The core idea is that individuals interpret psychologically distant events more abstractly. Previous studies have verified this by finding that when individuals are asked to consider issues from a more distant self-perspective, they demonstrate greater wise reasoning in conflicting situations (Grossmann and Kross 2014). This suggests that wisdom is not only about individuals transcending a narrow, self-centered perspective but also involves adopting a macro view and employing holistic reasoning strategies to grasp the bigger picture (Kross and Grossmann 2012; Staudinger and Glück 2011). Moreover, higher level of abstract construal facilitates the application of moral principles to psychologically distant behaviors (Eyal et al. 2008), thereby exemplifying wise behavior that seeks to balance the interests of all parties (Sternberg and Glück 2022). Yet wisdom extends beyond moral reasoning to include the motivation and courage to act ethically, even in the face of social pressures or personal costs. As Sternberg and Glück (2022) note, translating ethical insight into wise action requires problem recognition, perspective-taking, and emotion regulation. Mindfulness may serve as a catalyst for this process by heightening awareness, fostering reflective distance, and promoting value-consistent behavior. Importantly, wisdom is positively associated with life satisfaction and prosocial orientation, suggesting that cultivating mindfulness and wisdom together may not only enhance individual well-being but also promote actions that serve the common good. Finally, mindfulness interventions can also help individuals describe events with a higher level of abstract interpretation (Chan and Wang 2019), suggesting that CLT may serve as an effective mechanism linking mindfulness and wisdom.

### 1.3. Present Study

In summary, prior studies have primarily examined the short-term effects of mindfulness interventions on wisdom or established their relationship through cross-sectional designs. Yet little is known about how mindfulness, as a stable psychological trait, shapes wisdom development over time. To address this gap, the present study conducted a three-wave longitudinal survey with Chinese university students in early adulthood. This design enables us to trace the developmental trajectory of wisdom while assessing the predictive role of mindfulness beyond short-term changes. A random intercept cross-lagged panel model (RI-CLPM) was employed to separate between-person differences from within-person dynamics (Hamaker et al. 2015; Yu and Li 2023; Yuan et al. 2021). By modeling both stable individual characteristics and intra-individual processes, this approach allows for a more precise understanding of the dynamic interplay between mindfulness and wisdom across time.

## 2. Materials and Methods

### 2.1. Participants

This study conducted a priori power analysis for cross-lagged analysis using the powRICLPM package in R (Mulder 2022), assuming a power of 1 − *β* of 80% and an α level of 0.05. Given that this study involved three longitudinal repeated measures, the number of repeated measures was set at three, the intraclass correlation coefficient (ICC) for between-group variance was set at 0.3, and the autoregressive effect was set at a medium-small effect size of 0.3 (Cohen 1988). These parameters indicated that at least 600 participants were required to detect an autoregressive effect of *r* = 0.30. This study utilized cluster sampling to conduct a follow-up survey of first-year students from 11 classes at two universities starting in October 2023. The three measurement periods were October 2023 (T1, *N* = 782), November 2023 (T2, *N* = 792), and January 2024 (T3, *N* = 774). The second measurement included ten students who were transferred in, whereas the third measurement had 20 fewer participants than the second, resulting in a longitudinal attrition rate of 2.52%. Ultimately, 719 participants who completed all three surveys were included in the study (average age: 18.18 ± 0.67 [16–21]; 29.70% men; annual income: ¥12,000–24,000 [median]; social class: 4.03 ± 1.20 [1–7]; 93.18% the Han Chinese).

### 2.2. Procedures

The leading researcher for this assessment was a trained master’s psychology student. An online questionnaire was established on the Credamo platform, with each testing phase lasting one week. Reminders were sent at ten and six o’clock on the testing week’s first, third, and sixth days. The survey was concluded at six o’clock on the seventh day.

The specific process is as follows: first, mindfulness was measured; second, participants were asked to recall recent interpersonal conflict events (Grossmann and Kross 2014): they should imagine “a recent conflict situation,” and for each conflict event, participants were required to answer a series of questions to ensure the validity of the event reconstruction: (1) When did this conflict occur? (2) What day of the week did this occur? (3) At what time of day did it take place? (4) Where were you at the time? (5) Who individuals were involved in this study? (6) Was the person you had a conflict with the same gender as you? (7) Please describe this conflicting event in at least 70 words (Zhang et al. 2024); finally, we measured the participants’ wise reasoning and thinking randomly.

### 2.3. Measurements

#### 2.3.1. Five Facet Mindfulness Questionnaire

Mindfulness was measured by Chinese version of the Five Facet Mindfulness Questionnaire (FFMQ) (Deng et al. 2011), which consists of 39 items, such as “I can accurately express my emotional state in words.” These items encompass five dimensions: describing (describing with words), observing (observing and attending), acting with awareness (acting with awareness), non-judging of experience (non-judging of experience), and non-reactivity (non-reactivity). Each item is rated on a 5-point Likert scale from 1 (strongly disagree) to 5 (strongly agree). The reliability Cronbach’s *α*-coefficient for the three measurements in this study were 0.91, 0.93, and 0.95. The overall average score was used to indicate trait mindfulness, with higher scores indicating higher levels of trait mindfulness.

#### 2.3.2. Situated Wise Reasoning Scale

This study employed the Situated Wise Reasoning Scale (SWIS) developed by Brienza et al. (2018) to assess individuals’ capacity for wise reasoning. The Chinese version of the scale was downloaded from Grossmann’s web site (https://uwaterloo.ca/wisdom-and-culture-lab/measures, accessed on 15 December 2024). The scale includes items such as “I/my friends/strangers can empathize with others’ perspectives.” It comprises 21 items covering five core dimensions: recognition of others’ perspectives or broader contexts than the issue at hand, recognition of uncertainty and change, intellectual humility or recognition of the limits of one’s knowledge, integration of different opinions or preferences for compromise, and an outsider’s vantage point. Each item is rated on a five-point Likert scale ranging from 1 (not at all true) to 5 (very true). This study used the total average score as an indicator of wise reasoning, with higher scores indicating a higher level of wise reasoning. The reliability Cronbach’s *α*-coefficient for the three measurements were 0.96, 0.93, and 0.97.

#### 2.3.3. Wise Thinking Scale

This study employed the Wise Thinking Scale (WTS) developed by Fu et al. (2021), which is designed to evaluate Chinese individuals’ tendencies toward wise thinking in conflict resolution. This scale includes 14 items covering four dimensions: consideration from different perspectives, insight, benevolence, and recognition of uncertainty (e.g., “I/my friends/strangers hope to resolve conflicts among parties as much as possible”). Each item is rated on a six-point Likert scale (1 = not at all true to 6 = very true). The total average score on the scale indicates wise thinking, with higher scores indicating higher levels of wise thinking. Cronbach’s *α*-coefficient for the three measures were 0.95, 0.95, and 0.96, respectively.

#### 2.3.4. Demographic Information

Participants completed a demographic information survey including age, sex, highest education level, income, and subjective social status (the MacArthur Scale of subjective SES; Adler et al. 2000).

## 3. Results

### 3.1. Data Analyses

First, we preliminary examined the averages of mindfulness, wise thinking, and reasoning and the correlation coefficients among these variables over the three assessments (see Table 1).

Next, a longitudinal multi-level analysis (LMA) is employed to examine the trajectories of changes in each variable: (1) a simple random intercept model without predictor variables was established to estimate the mean, intraclass correlation coefficient (ICC), and variances within and between individuals; (2) the basic trajectories of changes in each variable were identified, using time and the quadratic term of time as predictor variables, to test for linear and curvilinear patterns over time (see Table 2).

Furthermore, a multi-level regression model was established to examine the impact of the initial level of mindfulness and changes in mindfulness on wisdom (see Table 3).

**Figure 1:**
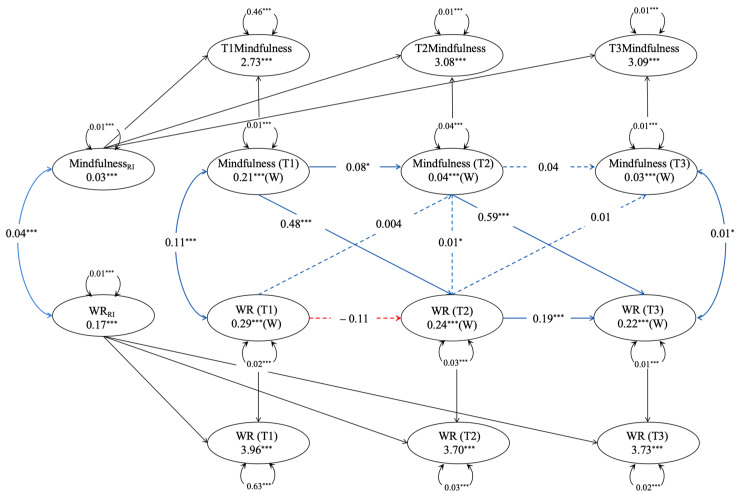
Random Intercept Cross-Lagged Model of Mindfulness and Wise Reasoning. * *p* < 0.05, *** *p* < 0.001; WR = Wise Reasoning. Solid lines indicate significant standardized path coefficients, and dashed lines indicate non-significant standardized path coefficients; blue lines represent positive values, and red lines represent negative values. RI = random intercept, W = within-person component. Same as below.

**Figure 2:**
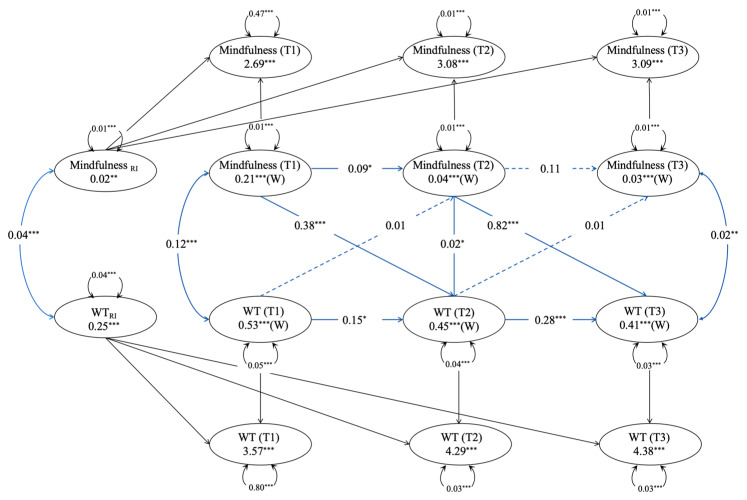
Random Intercept Cross-Lagged Model of Mindfulness and Wise Thinking. * *p* < 0.05, ** *p* < 0.01, *** *p* < 0.001; WT = Wise Thinking.

Finally, a random-intercept cross-lagged panel model (RI-CLPM) is constructed to explore the predictive relationship between mindfulness and wisdom (see Table 4 and Figure 1 and Figure 2).

### 3.2. Common Methods Variance

As the research employed self-report methods, it was susceptible to common method bias. Therefore, the questionnaires in this study were presented randomly, and different Likert scoring methods were established. Moreover, Harman’s single-factor test revealed that there were 12, 10, and 9 factors with eigenvalues greater than 1 in the three measurements. The variances explained by the first factor for each of the three measurements were 30.56%, 29.25%, and 33.54%, all below the critical threshold of 40%. This indicated no significant common method bias in the three measurements (Zhou and Long 2004).

### 3.3. Descriptive Analysis and Correlation Analysis

Table 1 presents each variable’s mean values, standard deviations, and correlation coefficients at three-time points. The results indicated significant positive correlations among the variables at all three-time points. The mean values for intelligent reasoning and thinking showed an increasing trend.

### 3.4. Trajectory of Mindfulness and Wisdom

#### 3.4.1. The Linear and Nonlinear Trajectory of Mindfulness and Wisdom

This study employed a longitudinal multi-level analysis to examine the trajectory of changes in mindfulness and wisdom. The results are presented in Table 2.

Mindfulness: (1) The intraclass correlation coefficient (ICC) for mindfulness is 0.608, indicating that 60.8% of the variance is due to differences between individuals; (2) the linear time model for mindfulness is not significant, with a likelihood ratio (LR) χ^2^(1) = 1.94, *p* = 0.166; the quadratic time model is also not significant, with a likelihood ratio (LR) χ^2^(1) = 0.02, *p* = 0.892. Therefore, trait mindfulness levels did not change over time.

Wise Reasoning: (1) The ICC for wisdom reasoning is 0.560, meaning that 56.00% of the variance is due to differences between individuals; (2) the linear time model for wisdom reasoning is significant, with a likelihood ratio (LR) χ^2^(1) = 7.63, *p* = 0.006; the quadratic time model is also significant, with a likelihood ratio (LR) χ^2^(1) = 5.32, *p* = 0.022.

Wise Thinking: (1) The ICC for wisdom thinking is 0.467, indicating that 46.7% of the variance is due to differences between individuals; (2) the linear time model for wisdom thinking is significant, with a likelihood ratio (LR) χ^2^(1) = 4.91, *p* = 0.028; the quadratic time model is not significant, with a likelihood ratio (LR) χ^2^(1) = 1.76, *p* = 0.286.

Therefore, only the linear model was retained as the final wisdom model (Table 3). The model estimates show (Table 3, Wisdom Reasoning Model 1) that the initial average level of wisdom reasoning is 3.67, which then improves over time (γ_10_ = 0.06, SE = 0.02, *p* = 0.006) and increases at an annual rate (as indicated by the quadratic effect of time, γ_20_ = −0.06, SE = 0.04, *p* = 0.022). There are significant individual differences in the initial level of wisdom reasoning (σ_00_ = 0.26, SE = 0.04, *p* < 0.001), and it changes linearly over time (σ_11_ = 0.04, SE = 0.01, *p* < 0.001). The covariance between the initial level of wisdom reasoning and its linear rate of change over time is also significant (σ_10_ = −0.04, SE = 0.01, *p* = 0.006), indicating that participants who exhibit a high level of wisdom reasoning in the initial phase (T1) tend to show slower growth in wisdom reasoning over time.

The model estimates (Table 3, Wisdom Thinking Model 1) indicate that the initial average level of wisdom thinking is 4.22, which also improves over time (γ_10_ = 0.06, SE = 0.03, *p* = 0.028) but does not increase at an annual rate (as indicated by the quadratic effect of time, γ_20_ = −0.06, SE = 0.05, *p* = 0.186). There is a significant individual difference in the initial level of wisdom thinking (σ_00_ = 0.30, SE = 0.06, *p* < 0.001), but no linear change over time (σ_11_ = 0.03, SE = 0.02, *p* = 0.463). The covariance between the initial level of wisdom thinking and its linear rate of change over time is also significant (σ_10_ = −0.02, SE = 0.04, *p* = 0.003). This indicates that for participants who exhibited a high level of wisdom thinking in the initial phase (T1), although their wisdom thinking improved over time, their growth was not significant.

#### 3.4.2. The Effect of Mindfulness on the Trajectory of Wisdom

This research also examined mindfulness’s inter- and intra-individual effects on the trajectory of wisdom change.

**Wise reasoning**: The initial level of mindfulness at T1 is significantly positively correlated with wisdom reasoning at T1 (γ_mindfulness_initial_ (B) = 0.49, SE = 0.12, *p* < 0.001), with no significant interaction effect over time (γ_mindfulness_initial_ (B) × Time = −0.01, SE = 0.06, *p* = 0.871). The analysis of intra-individual effects indicates that an increase in mindfulness level (relative to T1) is significantly positively correlated with higher wisdom reasoning levels at a given time point (γ_mindfulness_ (W) = 0.17, SE = 0.09, *p* = 0.056); that is, when individuals possess higher trait mindfulness levels (compared to the initial level), they also exhibit higher wisdom reasoning levels (see Table 3, Model 2).

**Wise thinking**: The initial level of mindfulness at T1 is not correlated with wisdom thinking at T1 (γ_mindfulness_initial_ (B) = 0.55, SE = 0.15, *p* < 0.001), with no significant interaction effect over time (γ_mindfulness_initial_ (B) * Time = 0.02, SE = 0.13, *p* = 0.848). The analysis of intra-individual effects shows that an increase in mindfulness level (relative to T1) is significantly positively correlated with higher wisdom thinking levels at a given time point (γ_mindfulness_ (W) = 0.03, SE = 0.13, *p* = 0.843). This indicates that when individuals have higher mindfulness levels (compared to the initial level), it does not lead to higher wisdom thinking levels (see Table 3, Model 2).

### 3.5. Random Intercept Cross-Lagged Analysis

The study utilized the R package lavaan (version 0.6-17, Rosseel 2012) to conduct a random intercept cross-lagged panel model (RI-CLPM, Hamaker et al. 2015) to distinguish between-person and within-person effects.

#### 3.5.1. Mindfulness and Wise Reasoning

This study constructed a random intercept cross-lagged mindfulness and wisdom reasoning model across three-time points. After systematically removing non-significant pathways, the final cross-lagged model exhibited good fit indices (Table 4). The results indicate that the random intercept for mindfulness (*β* = 0.02, *p* = 0.004) and for wisdom reasoning (*β* = 0.17, *p* < 0.001) significantly deviates from zero, suggesting inter-individual differences in mindfulness levels and wisdom reasoning among the study participants, warranting a cross-lagged analysis of random intercepts. Furthermore, the covariance relationship between the random intercepts of mindfulness and wisdom reasoning was significantly positive (*r* = 0.04, *p* < 0.001), indicating that as an individual’s mindfulness level increased, their wisdom reasoning level also increased. The results depicted in Figure 2 show that, after controlling for all demographic variables and social strata, mindfulness predicts wisdom reasoning (T1–T2: *β* = 0.48, *p* < 0.001; T2–T3: *β* = 0.59, *p* < 0.001), while wisdom reasoning cannot predict mindfulness in return (see Figure 1).

#### 3.5.2. Mindfulness and Wise Thinking

Similarly, the model fit indices of the cross-lagged model are good (see Table 4). The random intercept of wisdom thinking (*β* = 0.25, *p* < 0.001) significantly deviates from 0, indicating individual differences in wisdom thinking among the study participants, which justifies the need for a random intercept cross-lagged analysis. Furthermore, the covariance relationship between the random intercepts of mindfulness and wisdom thinking was significantly positive (*r* = 0.04, *p* < 0.001), suggesting that as individuals’ mindfulness levels improved, their wisdom thinking also increased. The results in Figure 2 show that after controlling for all demographic variables and social class, mindfulness predicts wisdom thinking (T1–T2: *β* = 0.38, *p* < 0.001; T2–T3: *β* = 0.82, *p* < 0.001), while wisdom thinking does not predict mindfulness in return (see Figure 2).

## 4. Discussion

During the transition from adolescence to early adulthood, individuals experience rapid development of cognitive abilities and thinking styles. Simultaneously, owing to the continuous accumulation of relevant knowledge and experience, the wisdom exhibited during this stage shows a significant accelerating trend (Jordan 2005). Based on the Growth Theory of wisdom and supported by related literature (e.g., Wang and Wang 2018), this study posits that wisdom during this period displays a specific linear growth trend and that short-term tracking is sufficient to effectively capture the initial developmental trajectory of wisdom and its short-term changes. Therefore, this study employed a short-term, longitudinal tracking design across three-time points to explore the developmental trajectory of wisdom in early adulthood and its predictive role in mindfulness.

### 4.1. Trajectory of Wise Reasoning and Thinking

A longitudinal multi-level analysis found that wise thinking and reasoning show linear growth during early adulthood. This finding may align with Sternberg and Glück’s (2022) theoretical framework, which emphasizes that wisdom is a multidimensional construct, encompassing analytical, creative, and practical abilities, as well as the capacity to balance personal, interpersonal, and higher-order interests. Early adulthood is a period of rapid accumulation of knowledge, professional experience, and social engagement, providing a solid foundation for wisdom development. Accordingly, the observed linear growth may primarily reflect the steady accumulation of cognitive and practical skills, whereas the development of ethical judgment and higher-order social responsibility may follow more complex, potentially nonlinear trajectories in middle and later adulthood.

Implicit theories of wisdom suggest that old age is one of the characteristics associated with wisdom (Clayton and Birren 1980), and those nominated as wisdom often exceed 50 years (Bluck and Glück 2005). However, previous findings on the relationship between age and wisdom have been inconsistent. Some studies have found a nonlinear relationship between age and wisdom, such as a positive quadratic relationship (Brienza et al. 2018), indicating that wisdom levels may initially rise and then decline with age, whereas others have revealed a negative quadratic relationship (Ardelt and Jeste 2018; Webster et al. 2014), suggesting that wisdom may peak in middle or late adulthood and then gradually decline. Additionally, some studies have demonstrated a positive correlation between age and wisdom (e.g., Grossmann et al. 2013), indicating that wisdom levels also improve with age; however, there are also findings suggesting a negative correlation (e.g., Ardelt 2016) or no correlation at all (e.g., Ardelt 2010) between age and wisdom. These discrepancies may be related to sample size, research design, or cultural background, leaving the age–wisdom relationship unresolved and warranting further investigation.

Longitudinal studies of wisdom across the entire lifespan are challenging to conduct, and past research has primarily relied on cross-sectional self-reports, which may not accurately capture the developmental trajectory of wisdom. Nevertheless, the period from adolescence to early adulthood is recognized as a nascent stage of wisdom development, characterized by accelerated improvements due to continuous knowledge accumulation (Jordan 2005) and steady annual increases (Sternberg 2005). In line with this, the present study selected college students in early adulthood as participants to examine the trajectory of wisdom development. The results confirmed that wisdom grows linearly over time during early adulthood, consistent with theoretical predictions (Wang and Wang 2018). Moreover, individual differences were observed: participants with higher initial wisdom levels showed slower growth over time, suggesting the presence of “early wisdom” versus “late bloomers” within the same cohort.

### 4.2. Effect of Mindfulness on Wise Reasoning and Thinking

Cross-lagged analysis with random intercepts revealed that mindfulness was positively correlated with wise thinking and wise reasoning. At the intra-individual level, mindfulness robustly predicted wise thinking and wise thinking. Trait mindfulness is a key element in cultivating wisdom and is closely related to moral principles (Amaro 2015; Baer 2015). Through moral practices, trait mindfulness helps reduce greed and aversion, diminishing the mind’s wandering and fostering mental tranquility. This tranquility aids individuals in self-regulation and self-transcendence, allowing for a profound understanding of the relationship between the self and the world, thus gaining insight into the essence of phenomena and advancing wisdom development (Wang et al. 2023; Verhaeghen 2019).

Wise individuals navigate problems with the right and novel paths, adhering to ethical guidelines as oars for problem-solving. It must also ensure that the outcomes of their actions neither infringe upon the legal rights of others and society nor, in the long term, fail to promote the enhancement and reinforcement of moral norms, thus contributing to the welfare of others and society and serving as an essential consideration in problem-solving (Wang and Fu 2017). Repeated measurements of individual mindfulness levels may serve as an indirect form of practice. Mindfulness can positively influence the development of wisdom through various mechanisms, including physiological and psychological benefits. First, practitioners’ non-judgmental awareness of current thoughts and emotions can effectively reduce biases, encouraging individuals to remain open to experiences (Lindsay and Creswell 2017); second, mindfulness practice can assist individuals in understanding, regulating, and reducing immature responses to emotions. Furthermore, mindfulness practice can help practitioners enhance their reflective and emotional regulation abilities and experience and gain insights into the essence of thoughts from a de-centered perspective (Safran and Segal 1990).

Through these mechanisms, individuals gradually improve psychological distance regulation, increase their tendencies toward self-detachment, and reduce rumination and negative emotions (Kabat-Zinn 2003; Sauer and Baer 2012; Wang et al. 2024; Zhang et al. 2017). Consequently, they can examine problems from a broader perspective, avoiding the trap of narrow, self-centered thinking and thereby demonstrating greater wisdom in complex decision-making contexts (Wang et al. 2022).

### 4.3. Significance

First, it validated the Growth Theory of wisdom (Jordan 2005) through short-term tracking of early adult college students, indicating that wisdom increases linearly over time during early adulthood. However, this study also found that individuals with initially higher levels of wisdom and wisdom thinking exhibit slower growth rates. This finding expands upon implicit theories of wisdom regarding the relationship between wisdom and age. It fills a gap in longitudinal research, providing a new perspective on the dynamic development of wisdom and its time-varying patterns. Although this conclusion currently applies only to the early adult population, it offers an essential theoretical foundation and practical guidance for future studies on wisdom across different age groups.

Second, this study confirmed the predictive role of trait mindfulness in wisdom reasoning and thinking, providing reliable, supportive evidence for the theory of mindfulness in the development of self-wisdom (Karunamuni and Weerasekera 2019). Mindfulness is rooted in Buddhist meditation, where the ultimate goal of Buddhist practice is to cultivate self-knowledge and wisdom, with wisdom defined as “right understanding” or a thorough comprehension of the essence of phenomena (Anālayo 2006). The key elements of Buddhist practice include cultivating ethics and compassion, training the mind for tranquility to reduce distractions, and ultimately developing wisdom (Ñanamoli and Bodhi 1994). Findings on the mindfulness prediction of wisdom further deepen the moral foundation theory of the mechanisms related to mindfulness and wisdom, which stems from the intrinsic connection between mindfulness and morality in Buddhist practices (Norenzayan 2013). Some empirical studies also support that mindfulness promotes prosocial behavior, particularly in fostering empathy and increasing prosocial actions (e.g., Hafenbrack et al. 2020; Poulin et al. 2021). These studies provide a more valuable understanding of moral pathways (Wang et al. 2024) and the longitudinal predictive relationships of mindfulness in predicting wisdom.

Third, the core mechanism of mindfulness enhances the ability to regulate self-psychological distance (Kabat-Zinn 2003), consistent with the predictive and explanatory level theory (CLT) of wisdom and wisdom thinking. Both wisdom reasoning and wisdom thinking emphasize recognition of others’ perspectives/broader contexts than the issue at hand (Fu et al. 2021; Grossmann 2017), while an increase in self-psychological distance helps individuals describe events from a more global, abstract, and expansive perspective (Trope and Liberman 2010). Openness to experience, one of the core dimensions of mindfulness, encourages abstract thinking (Chan and Wang 2019). This enriches the explanatory level theory and develops and supplements the model of mindfulness predicting wisdom based on that theory.

Finally, our findings not only advance our understanding of the dynamic interplay between mindfulness and wisdom but also have important implications for developmental psychology and educational practices, highlighting how cultivating mindfulness may support adolescents’ wisdom and moral growth. Building on the understanding that mindfulness fosters wisdom by promoting the observation of one’s thoughts and behaviors, a mindfulness-based intervention may be particularly beneficial for the juvenile population. The program would focus on training participants to observe their internal experiences—including thoughts, emotions, and impulses—without immediate judgment. Through guided exercises such as breath awareness, body scans, and reflective journaling, adolescents would learn to pause and respond thoughtfully to situations rather than react impulsively. By reducing automatic, emotionally driven responses, the intervention aims to enhance self-regulation, perspective-taking, and deliberate reflection—key components of wise reasoning. The program could be implemented in school or community settings over several weeks, combining structured sessions with daily mindfulness practices. Outcomes would be evaluated by tracking changes in mindfulness, impulsivity, and wisdom-related behaviors, providing insight into the effectiveness of cultivating wisdom through mindful observation in adolescents.

## 5. Limitations and Future Directions

This study expands longitudinal research on mindfulness and wisdom, deepening and enriching the relevant mechanisms and theories predicting wisdom through mindfulness. However, this study has some limitations. (1) this study’s relatively short tracking period made it challenging to infer the long-term characteristics of wisdom development or the long-term predictive relationship pattern between mindfulness and wisdom. The development of wisdom requires long-term assimilation and reflection on life experiences (Wang et al. 2024). Short-term studies are insufficient to reveal clear pathways in this process. Considering age as an essential characteristic of wisdom (Clayton and Birren 1980), short-term studies may struggle to capture its deeper influence mechanisms. Therefore, future research should adopt a longitudinal tracking design extending from early adolescence to adulthood to examine the developmental trajectory of wisdom and explore the mechanisms by which mindfulness affects wisdom development over a more extended period. (2) Although this study employed two different measurement methods for wisdom, the results indicated that the measurement method did not significantly impact the research conclusions. Both methods were self-reported measures. Furthermore, this study exclusively utilized self-assessment in interpersonal conflict situations—which, while providing an in-depth social context for wisdom research, may not fully encompass the rich connotations of wisdom. As wisdom is a complex, multidimensional concept, future studies could incorporate more diverse measurement approaches, including multi-context measurements (such as integrating various types of situational questions like life challenges and ethical dilemmas) to broaden the scope of research on wisdom reasoning and thinking; multi-method assessments, using evaluations from others and performance-based measurement methods to compensate for potential subjective biases in self-reports; cross-cultural research that integrates the characteristics of wisdom theories in different cultural contexts, particularly between Eastern and Western cultures, to conduct a more comprehensive exploration of the connotation of wisdom; and employing text analysis techniques to mine large samples or big data to establish situational databases suitable for different age groups, providing richer material for qualitative wisdom research.

## 6. Conclusions

This study conducted a longitudinal tracking of university students in early adulthood, and the results indicated that the wisdom level of students at this stage showed a linear growth trend. The random intercept cross-lagged analysis further revealed that mindfulness positively predicted wisdom. This result is consistent across wisdom-related indicators (e.g., wise reasoning and wise thinking). It provides empirical support and practical guidance for cultivating and developing wisdom among university students.

## Figures and Tables

**Table 1:** Means and Correlations for Variables Across Three Timepoints.

Variables	M	SD	1	2	3	4	5	6	7	8
Mindfulness (T1)	3.10	0.49	—							
Mindfulness (T2)	3.08	0.26	0.37 ***	—						
Mindfulness (T3)	3.09	0.23	0.29 ***	0.48 ***	—					
Wise Reasoning (T1)	3.65	0.70	0.48 ***	0.26 ***	0.22 ***	—				
Wise Reasoning (T2)	3.70	0.67	0.41 ***	0.31 ***	0.23 ***	0.44 ***	—			
Wise Reasoning (T3)	3.73	0.65	0.32 ***	0.38 ***	0.31 ***	0.47 ***	0.55 ***	—		
Wise Thinking (T1)	4.26	0.90	0.43 ***	0.26 ***	0.21 ***	0.82 ***	0.44 ***	0.47 ***	—	
Wise Thinking (T2)	4.29	0.87	0.38 ***	0.32 ***	0.24 ***	0.47 ***	0.78 ***	0.54 ***	0.51 ***	—
Wise Thinking (T3)	4.38	0.87	0.34 ***	0.40 ***	0.34 ***	0.39 ***	0.53 ***	0.81 ***	0.46 ***	0.56 ***

*** *p* < 0.001.

**Table 2:** Equations for Linear, Quadratic Model for Basic Trajectory.

	Estimate (SE)			Model Fit		
Model	Intercept	Time	Time2	−2LL	AIC	BIC
Mindfulness						
Linear model	3.05 (0.05) ***	0.01 (0.01)		11.59	23.59	50.32
Quadratic Model	3.05 (0.02) ***	0.01 (0.03)	0.00 (0.01)	11.57	25.57	56.75
Wise Reasoning						
Linear model	3.61 (0.04) ***	0.06 (0.03) **		924.95	936.95	963.69
Quadratic Model	3.56 (0.04) ***	0.19 (0.08) *	−0.06 (0.03) *	919.70	933.70	964.89
Wise Thinking						
Linear model	4.16 (0.05) ***	0.06 (0.04) *		1338.59	1350.59	1377.32
Quadratic Model	4.14 (0.05) ***	0.18 (0.09) ^+^	−0.06 (0.05)	1336.84	1350.84	1382.02

^+^ *p* < 0.1, * *p* < 0.05, ** *p* < 0.01, *** *p* < 0.001.

**Table 3:** Model Estimates for the Change in Wisdom During Emerging Adulthood With Time-Invariant and Time-Varying Covariates.

	Model 0		Model 1		Model 2	
Fixed Effect	Estimate (SE)	95% CI	Estimate (SE)	95% CI	Estimate (SE)	95% CI
Wise Reasoning						
Intercept	3.67 (0.03) ***	[3.599, 3.7343]	3.61 (0.04) ***	[3.528, 3.688]	3.61 (0.04) ***	[3.531, 3.685]
Time			0.06 (0.01) ***	[0.017, 0.100]	0.06 (0.02) **	[0.015, 0.098]
Mindfulness (B)					0.49 (0.12) ***	[0.253, 0.720]
Mindfulness (B) × Time					−0.01 (0.06)	[−0.137, 0.116]
Mindfulness (W)					0.17 (0.09) ^+^	[−0.005, 0.351]
Random Effect						
σ_00_: Intercept variance	0.20 (0.02) ***	[0.154, 0.251]	0.26 (0.04) ***	[0.195, 0.334]	0.23 (0.03) ***	[0.176, 0.306]
σ_11_: Slope variance			−0.04 (0.02) **	[−0.072, −0.012]	0.04 (0.01) ***	[0.023.0.067]
ε: Residual variance	0.15 (0.01) ***	[0.135, 0.176]	0.11 (0.01) ***	[0.092, 0.135]	0.11 (0.01) ***	[0.090, 0.131]
Wise Thinking						
Intercept	4.22 (0.03) ***	[4.138, 4.306]	4.16 (0.05) ***	[4.060, 4.259]	4.16 (0.05) ***	[4.064, 4.256]
Time			0.06 (0.03) ***	[0.007, 0.118]	0.06 (0.03) *	[0.006, 0.118]
Mindfulness (B)					0.55 (0.15) ***	[0.262, 0.845]
Mindfulness (B) × Time					0.02 (0.09)	[−0.153, 0.186]
Mindfulness (W)					0.03 (0.13)	[−0.230, 0.281]
Random Effect						
σ_00_: Intercept variance	0.28 (0.04) ***	[0.212, 0.362]	0.30 (0.06) ***	[0.205, 0.434]	0.27 (0.04) ***	[0.178, 0.397]
σ_11_: Slope variance			0.03 (0.02)	[0.006, 0.128]	0.04 (0.01) ***	[0.023, 0.067]
ε: Residual variance	0.32 (0.01) ***	[0.276, 0.362]	0.29 (0.02) ***	[0.237, 0.347]	0.29 (0.03) ***	[0.236, 0.346]

^+^ *p* < 0.1, * *p* < 0.05, ** *p* < 0.01, *** *p* < 0.001. B = between person, W = within person; Model 0 is an unconditional model estimating the intraclass correlation coefficient; Model 1 estimates the basic trajectory of wisdom over time; Model 2 estimates the between-individual (time-invariant) and within-individual (time-varying) effects of mindfulness levels on the trajectory of wisdom.

**Table 4:** Model Fit Indices for the Random Intercept Cross-Lagged Model of Mindfulness and Wisdom.

Model	Variables	Intercept (SE)	Model Fit				
			χ^2^/*df*	CFI	TLI	SRMR	RMSEA [90% CI]
1	Mindfulness	0.03 (0.01) ***	30.34 ***	0.947	0.879	0.055	0.070 [0.055, 0.087]
	Wise Reasoning	0.17 (0.02) ***					
2	Mindfulness	0.02 (0.01) **	30.19 ***	0.949	0.884	0.055	0.069 [0.053, 0.085]
	Wise Thinking	0.25 (0.04) ***					

** *p* < 0.01, *** *p* < 0.001.

## Data Availability

The original data presented in the study are openly available in https://osf.io/9avru/?view_only=b7e2a9aabd644364b0cc358bd7d5b88d.
